# The Intercontinental Terminals Chemical Fire Study: A Rapid Response to an Industrial Disaster to Address Resident Concerns in Deer Park, Texas

**DOI:** 10.3390/ijerph17030986

**Published:** 2020-02-05

**Authors:** Heyreoun An Han, Inkyu Han, Sheryl McCurdy, Kristina Whitworth, George Delclos, Amal Rammah, Elaine Symanski

**Affiliations:** 1 Southwest Center for Occupational and Environmental Health, Department of Epidemiology, Human Genetics and Environmental Sciences, The University of Texas Health Science Center at Houston (UTHealth) School of Public Health, 1200 Pressler, Houston, TX 77030, USA; Heyreoun.anhan@uth.tmc.edu (H.A.H.); Inkyu.han@uth.tmc.edu (I.H.); Amal.rammah@uth.tmc.edu (A.R.); 2 Center for Health Promotion and Prevention Research, Department of Behavioral Sciences and Health Promotion, UTHealth School of Public Health, Houston, TX 77030 USA; Sheryl.mccurdy@uth.tmc.edu; 3 UTHealth School of Public Health in San Antonio, 7411 John Smith, San Antonio, TX 78229, USA; 4 Center for Precision Environmental Health, Department of Medicine, Baylor College of Medicine, One Baylor Plaza, Houston, TX 77030, USA

**Keywords:** disaster response, ITC fire, air pollution, volatile organic compounds (VOCs), benzene, particulate matter (PM), black carbon (BC), health surveys

## Abstract

On Sunday, 17 March 2019, a fire erupted at the Intercontinental Terminals Company (ITC, Deer Park, La Porte, TX, USA), resulting in a large fire that blazed for several days. In response, we rapidly launched disaster response activities to monitor air pollutants (total volatile organic compounds (TVOCs), fine particulate matter (PM_2.5_), black carbon (BC), and ultra-fine particles (UFPs) during the fire in two affected communities. To assess immediate health effects and residential air quality, we also rapidly launched a pilot study, the Deer Park Chemical Fire (DeeP Fire) Study, in which we administered health surveys and installed samplers to monitor air quality outdoors of resident homes for up to six weeks. In both communities, mean ambient concentrations of PM_2.5_, BC and TVOCs were higher during the first week of the fire than a week after it was extinguished. Thirteen residents participated in the DeeP Fire Study. Most residents reported experiencing respiratory symptoms and some reported being bothered by at least one post-traumatic stress disorder symptom during the fire and two weeks afterwards. In the months following the fire, the 7-day mean ambient concentration of benzene from 12 homes was 0.13 ± 0.10 parts per billion (ppb) and the 6-week mean ambient concentrations of PM_2.5_ and TVOCs were 13 ± 6 µg/m^3^ and 108 ± 98 ppb, respectively. All residents requested and received individualized air monitoring reports. Surveillance systems that enable real-time monitoring of the environmental health impact during a major industrial incident are needed to provide timely information to adequately respond to a disaster in the future.

## 1. Introduction

**Figure 1:**
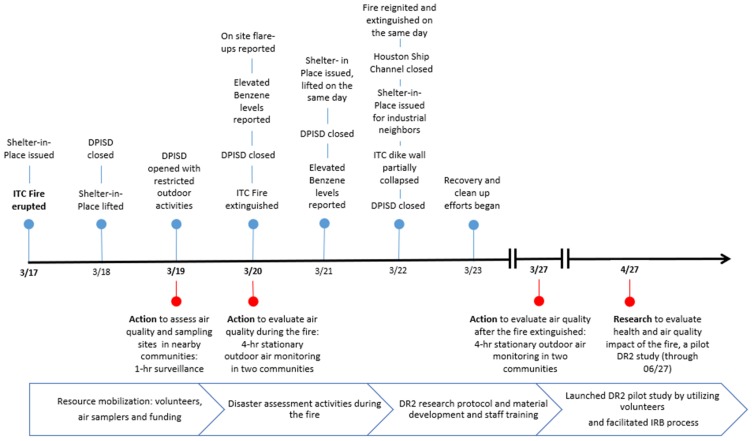
Timeline of ITC fire and action to research disaster response mechanisms. DPISD: Deer Park Independent School District; DeeP Fire Study: Deer Park Chemical Fire Study; ITC: Intercontinental Terminals Chemical; and DR2: disaster research response.

On Sunday, 17 March 2019, at approximately 10 a.m., a storage tank containing naphtha caught fire at the Intercontinental Terminals Company (ITC) Deer Park facility, a storage facility for petroleum liquids and gases operating in Deer Park, Texas, since 1972 (see Figure 1). The fire spread to other tanks over the ensuing six days [1], during which a blackened plume including toxic gases (e.g., benzene) and particulate matter (PM) traveled from Deer Park, Texas (TX) to other surrounding communities. Within five days of the fire, schools were canceled and several Shelter-In-Place orders were issued for Deer Park and nearby communities, raising concerns about the environmental health impacts of the fire [2]. Of concern was the potential for increased exposures to outdoor air pollution, including particulate matter (PM) and volatile organic compounds (VOCs) like benzene, toluene, ethylbenzene and xylenes (BTEX) during the fire. These concerns were heightened when the fire re-ignited, leading to a containment wall breach that released chemicals into the Houston Ship Channel [3]. In response, we mobilized resources to rapidly conduct community air monitoring and launched a disaster research response (DR2), the Deer Park Chemical Fire (DeeP Fire) pilot study. In this study, we evaluated the impact of the fire on the health of residents living in a neighborhood close to the ITC facility and conducted air monitoring outside their homes for 6 weeks. The pilot study tested DR2 methods that utilized volunteers, a facilitated Institutional Review Board (IRB) review process, and real-time, low-cost air sensors. This brief report aims to describe our efforts to rapidly respond and communicate the findings from our community air monitoring during the fire and the DeeP Fire study.

## 2. Materials and Methods

### 2.1. Community Description

**Figure 2:**
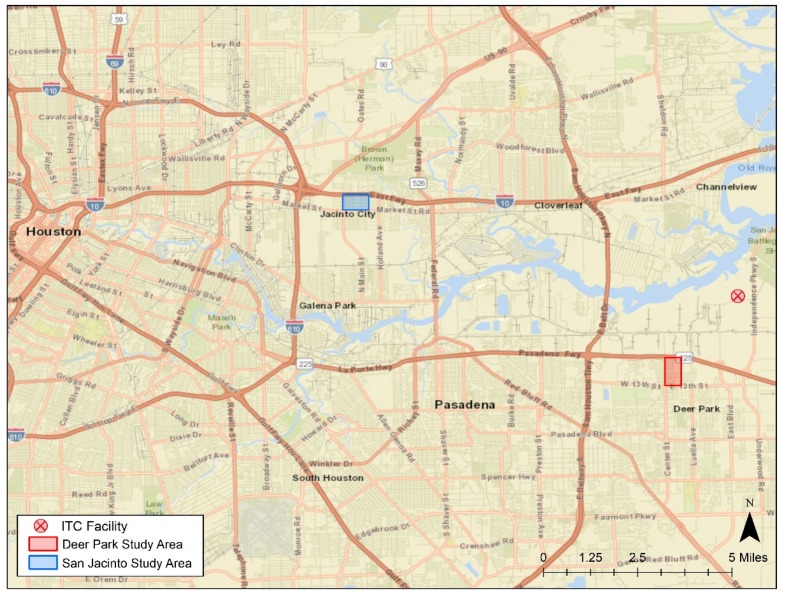
Two affected residential communities monitored during the ITC fire.

Our first response activities focused on two communities near the ITC facility: Deer Park and Jacinto City. Deer Park is located within three miles southwest of ITC and is approximately 20 miles east of Houston. Jacinto City, where the black smoke plume traveled while the fire burned, is about 10 miles northwest of ITC and 8 miles east of Houston, surrounded by major highways and railroads (See Figure 2). Both cities are predominantly white, with more residents identifying as Hispanic in Jacinto City than in Deer Park (76% versus 33%, respectively) [4]. In Deer Park, about 12% of the population has an annual household income of $25,000 or less while Jacinto City is more socioeconomically disadvantaged, with approximately 46% of the population earning $25,000 or less in annual household income.

### 2.2. Community Air Monitoring in Deer Park and Jacinto City During the Fire

During the fire and the five days after it was extinguished, we rapidly launched community air sampling to monitor a suite of air pollutants in two communities (see Figure 1). On 19 March, while the fire was spreading to multiple storage tanks, we surveilled the surrounding neighborhoods near the facility using handheld real-time air sensors to assess air quality and determine the fixed sampling locations for the ensuing days (e.g., empty curbside lot or school parking lot). On 20 and 27 March, we conducted about four hours of real-time air monitoring in Deer Park and Jacinto City, measuring total volatile organic compounds (TVOCs), fine particulate matter (PM_2.5_), black carbon (BC), and ultra-fine particles (UFPs) using a ppbRAE (RAE Systems, San Jose, CA, USA), a Grimm 11-R (GRIMM Aerosol Technik, Ainring, Germany), an AE51 (Aethlabs, San Francisco, CA, USA) and a Condensation Particle Counter (CPC) 3007 (TSI Inc., Shoreview, MN, USA), respectively, with 1-min logging intervals except for BC (5-min interval). The ppbRAE is a compact handheld monitor and datalogger for monitoring TVOCs using a photoionization detector with a 10.6 eV UV-discharge lamp. The Grimm 11-R is a handheld laser optic spectrometer to monitor particles ranging from 0.25 to 32 µm into 31 size bins. We report PM_2.5_ mass concentrations (size bin ≤2.5 µm) in this study. The AE51 is a pocket-size device measuring aerosol BC with an absorbance of wavelength at 880 nm. The CPC 3007 monitors number of particles using a laser light source, with a size range of 0.01 to >1.0 µm. These handheld direct reading instruments were fully charged overnight before field deployment. Following the manufacturer’s protocols, all operating parameters (e.g., flow rates and zero calibration) were checked before sampling began. Sampling was conducted simultaneously in two communities and all sampling devices were placed on folding tables, approximately 1.2 m from ground level.

### 2.3. DR2 Pilot Study in Deer Park a Month after the Fire

We issued a wide call for volunteers and launched the DeeP Fire Study to assess immediate environmental health effects following the fire. The DeeP Fire Study consisted of an interviewer-administered health survey and residential outdoor air monitoring. Because of limited resources and limited bilingual field staff, we focused this study on Deer Park and defined a buffer zone of approximately 300 households in a residential area closest to the facility. To recruit participants into the study, we canvassed the neighborhood door-to-door and distributed flyers describing the study (with the primary objectives of administering a health survey and conducting air sampling outside the home) and participation eligibility criteria (residents living in the defined buffer area, ≥18 years old). The flyers included study contact information and instructed residents to contact us by phone if they were interested in participating. Within three days of distributing the flyers, 22 residents left messages expressing interest. Of these individuals, 14 (64%) responded to our call-backs and we scheduled 13 (59%) home visits. During the call-back, we explained the purpose of the study and what participation would entail, including outdoor air monitoring and answering questions about the health impacts of the fire. We also indicated that participants who completed the air sampling would receive an individualized air monitoring report at the end of the study. We scheduled a home visit with participants who verbally confirmed their interest in participating and we sent reminders via email and/or text shortly before and on the day of the home visit. During the first home visit, written informed consent was obtained by trained field staff. The DeeP Fire Study was approved by the UTHealth Institutional Review Board (IRB# HSC-SPH-19-0241). All volunteer field staff completed human subject research ethics training and received field training on administering informed consent and the health survey, as well as on installing air sampling devices.

#### 2.3.1. Residential Air Monitoring to Collect Baseline Data a Month after the Fire

We conducted residential outdoor air monitoring from 27 April 2019 to 12 June 2019. After obtaining informed consent, the field team staff installed air sampling devices outside each participant’s home. We deployed two sampling devices: an electric-power operated real-time sensor (RESCUE, Academia Sinica, Taipei, Taiwan), which monitored TVOCs and PM_2.5_ with 5-min logging intervals for 6 weeks, and a passive diffusive sampler (3M Organic Vapor Monitor (OVM), 3M, Maplewood, MN, USA), which was deployed for 7 days for benzene, toluene, ethylbenzene, and xylene (BTEX) speciation. The RESCUE sensor device includes a light scattering laser and a metal oxide sensor for measurements of PM_2.5_ (resolution: 1 µg/m^3^) and TVOCs (resolution: 1 ppb), respectively. The air monitoring devices were located on the front porch or the patio on the backside of the house using a sampling stand near a power outlet (for the RESCUE sensor), if available. The OVM sensors were deployed at all 13 homes, whereas the RESCUE sensors were deployed at 9 homes where an outdoor power outlet was available. In total, we collected OVM samples at 12 residences and retrieved the RESCUE sensors at 8 residences as we were unable to retrieve the air sampling devices at one residence. All sampling devices were transferred to the laboratory and processed for further analyses. BTEX compounds extracted from the OVM samples were analyzed using an Agilent 6890 gas chromatography with a 5973 mass spectrometer (Agilent Technologies, Palo Alto, CA, USA) [5]. The field blank samples were used to determine method detection limits. All logged TVOCs and PM_2.5_ data were downloaded from the RESCUE sensors to a password-encrypted computer and validated. There was no gravimetric adjustment of recorded concentrations of PM_2.5_. For the community air monitoring data collected during the fire, we compared mean pollutant values between sampling days using a *t*-test, evaluated at a significance level of 0.05; for the residential air monitoring data, we generated descriptive statistics (mean, standard deviation, and median values). All analyses were performed using R (version 3.5.0, Vienna, Austria).

#### 2.3.2. Health Survey to Assess Health Effects During and Two Weeks After the Fire

Between 26 April 26 and 4 May, we administered a survey during the first home visit to one adult member from each of the 13 participating households. The survey consisted of 56 questions on demographics, medical history, and perceived physical and mental health during and two weeks after the fire. We also asked residents about their interest in receiving their residential air monitoring results and participating in future studies. The questions concerning physical health addressed general health conditions diagnosed before the fire (hay fever/seasonal allergies, asthma, emphysema or chronic obstructive pulmonary disorder, hypertension, diabetes, heart disease, migraines or frequent headaches, post-traumatic stress disorder (PTSD), kidney disease and insomnia or sleeping disorders), along with acute symptoms that may be attributed to the fire (shortness of breath, wheezing, persistent cough, nose irritation, chest pains, eye irritation, throat irritation, skin rash, frequent or severe headaches, heartburn or acid reflux, drowsiness and dizziness).

Mental health questions on the survey focused on symptoms relevant to PTSD, as well as depression and anxiety. Specifically, we adopted the World Health Organization (WHO) International Classification of Diseases, 11th version (ICD-11) Trauma Questionnaire (TQ), a self-report measure of the PTSD diagnosis [6]. The ICD-11 PTSD consists of 11 questions concerning three symptom clusters, including (1) re-experiencing of the event (RE; “having upsetting dreams”, “having powerful images or memories”, and “feeling very upset with the remainder of the experience”); (2) avoidance of the event reminder (AV; “avoiding internal reminders of the event” and “avoiding external reminders”); and (3) a persistent sense of threat (TH); “being super-alert” and “feeling jumpy”). Symptom endorsement score is on a Likert scale ranging from 0 to 4 (“not at all”, “a little”, “moderately”, quite a bit”, and “extremely”) in response to the question “*how much you were bothered by the problem during and two weeks after the fire*?”. A diagnosis of ICD-11 PTSD requires a score of ≥2 (“moderately”) for at least one symptom in each of three clusters [7]. In addition, to questions about depression, we used the Patient Health Questionnaire-8 (PHQ-8), a validated measure including 8 of the 9 questions in the PHQ-9 [8]. Possible responses included four categories asking the number of days the respondent had experienced symptoms during the two weeks after the fire: “not at all” = 0 to 1 day; “some days” = 2 to 6 days; “more than half the days” = 7 to 11 days; and “nearly every day” = 12 to 14 days, and points were assigned from 0 to 3, respectively [9,10,11]. The sum score of PTSD symptoms could range between 0 to 24 points: “no significant depressive symptoms” = 0 to 4; “mild depressive symptoms” = 5 to 9; “moderately severe” = 10 to 19; and “severe” = 20 to 24. The PHQ-8 cut-point of ≥10 and PHQ-8 algorithm diagnosis of major depression were used to classify major depression [8,10,12]. Additionally, respondents who experienced any of the PHQ -8 symptoms at least on “some days” were further asked to rate the impact of the fire on difficulties in their daily life at home and at work, and in relationships with other people on a scale of “not difficult at all”, “somewhat difficult”, “very difficult”, and “extremely difficult”.

## 3. Results

### 3.1. Impact of the ITC Fire on Community Air Quality

Table 1 shows the outdoor monitoring results in Deer Park and Jacinto City while the ITC facility was burning and five days after the fire was extinguished. Ambient air levels of TVOCs were 42%–73% higher in Deer Park while levels of PM_2.5_ (37%), BC (41%–45%) and UFP (2–8 times) were higher in Jacinto City. In both neighborhoods, levels of air pollutants were at least two times lower a week after the fire was extinguished, except for UFP concentrations in Jacinto City. The differences in mean air pollutant levels between 20 March and 27 March were significantly different (*p* < 0.05) at both locations.

### 3.2. DeeP Fire Pilot Study Findings

In total, 13 Deer Park residents enrolled in our study and completed the health survey. All respondents had completed more than a high school education (100%) with an average age of 53 years. Most respondents were female (92%), non-Hispanic whites (69%), non-smokers (85%), unemployed (62%), and had lived in their neighborhoods for more than five years (69%). The most-reported medical condition diagnosed before the fire was seasonal allergies (54%), followed by migraines (38%), hypertension (31%), asthma (23%), heart disease (23%), kidney disease (23%), and PTSD (8%). During the ITC fire and in the two weeks following, more than half of respondents experienced throat irritation (69%), persistent coughing (54%), and nose and eye irritation (54%), and approximately less than half of them consulted a doctor about their symptoms. The prevalence of ICD-11 PTSD was 31%, four out of the 13 respondents reported being “moderately” bothered by at least one symptom in each of the RE, AV, and TH symptom clusters. The most commonly reported PTSD symptoms were “being super-alert, watchful or on guard” (77%) in the TH cluster and “feeling upset when reminded of the ITC fire” (54%) in the RE cluster. Using the PHQ-8 cut scores of ≥10 for depression severity, nine respondents (62%) indicated no significant depressive symptoms (PHQ-8 scores = 1 to 4); three (23%) reported at least mild depressive symptoms (PHQ-8 scores = 5 to 9); and only one reported at least “moderately” depressive symptoms (PHQ-8 scores = 10 to 14), which typically represents significant depression regardless of diagnostic status with 88% sensitivity and 88% specificity for major depression [13]. Additional analysis following the major PHQ-8 depression algorithm [10] indicated that only one participant met the criteria for a provisional diagnosis of depression. Of the 12 individuals who experienced one or more depressive symptoms, seven (58%) indicated difficulty “getting along with other people” and “taking care of things at work and home”.

**Figure 3:**
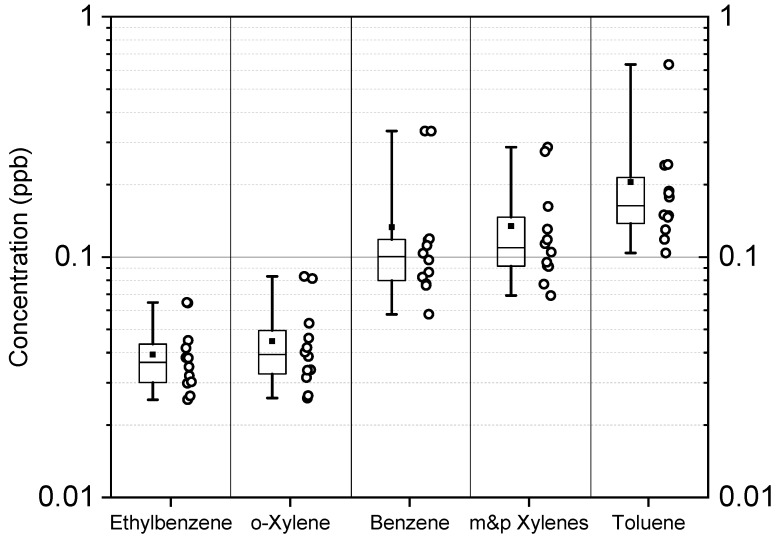
Box plots for individual residence BTEX (benzene, toluene, ethylbenzene, xylenes) concentrations. Open circles are BTEX concentrations in ppb. Horizontal lines in the box plot represent median values and filled squares represent mean values for each compound. Minimum and maximum concentrations are represented with whiskers.

The ambient air concentrations of PM_2.5_ and TVOCs that were measured outside participants’ homes are summarized in Table 2. Daily average PM_2.5_ concentrations ranged from 3 to 28 µg/m^3^ and the mean ± SD concentration was 13 ± 6 µg/m^3^. Mean concentrations of daily TVOCs were 108 ± 98 ppb (range: 17–536 ppb). Figure 3 shows the mean residential concentrations of benzene were 0.13 ± 0.10 ppb (median = 0.10 ppb) with a maximum concentration of 0.33 ppb at two houses. All residents expressed interest in receiving air monitoring results and received individual reports, in which we provided 7-day average concentrations of BTEX, average concentrations of TVOCs and 24-h averaged PM_2.5_ over the six weeks. For reference, we also included the air monitoring comparisons values (AMCV) for BTEX from the Texas Commission on Environmental Quality (TCEQ), the state’s environmental health agency, and the U.S. EPA’s National Ambient Air Quality Standard for PM_2.5_ [14].

## 4. Discussion

During the ITC fire, the burning tanks of naphtha and other gasoline compounds sent a plume of dark smoke over the Houston area for days and raised concerns about air quality and potential health impacts for residents in the surrounding area. Of concern was potentially increased exposures to PM and VOCs, like benzene, from both the 6-day fire and off-gassing after the suppression of the fire. Hence, we rapidly mobilized institutional resources to monitor air quality during the fire and conducted a DR2 pilot study in the following months to assess the environmental health impact of the ITC fire on a nearby community. To address environmental health concerns raised amongst affected residents, we provided individual air monitoring reports to study participants. During the first week of the fire, we detected concentrations of PM_2.5_, BC and UFPs in Jacinto City that were 37%, 45%, and 100% greater than in Deer Park, respectively. Given that the prevailing wind direction was from the southeast, higher concentrations were expected in the downwind location [15,16,17]. Further, seven days after the fire was extinguished, concentrations of most air pollutants significantly decreased by about 33% to 50% in both locations.

Concentrations of PM_2.5_ and TVOCs obtained from Grimm 11-R and ppbRAE devices should not be directly compared with measurements obtained using a federal reference method (FRM) such as gravimetric or gas chromatography methods. For instance, concentrations of PM_2.5_ from direct reading instruments that rely on light scattering are 2–3 times higher than FRM or FRM equivalent gravimetric methods [18,19,20]. Nonetheless, our results suggest that levels of air pollutants during the fire were significantly greater than after fire, indicating air quality in these communities was impacted by the fire, which is similar to assessments made following wildfires [21], the World Trade Center Disaster [22], and firework displays [23,24]. While it is unclear why UFP was increased in Jacinto City on 27 March, possible contributing sources may include a high volume of diesel truck traffic on I-10 and I-610 (major highways), as well as railway traffic and other nearby industrial operations.

To assess the environmental health impact of a natural or industrial disaster, a pre-disaster baseline assessment of air, soil, and water quality is needed, so that pre- and post-disaster comparisons can be made. Unfortunately, such data were not available for communities affected by the ITC fire. In our investigation about one month after the ITC fire, we found that 7-day mean concentrations of BTEX outside the homes of residents were less than 1 ppb (0.13 ppb). Except for ethylbenzene, the mean BTEX concentrations (benzene—0.13 ppb; ethylbenzene—0.04 ppb; toluene—0.21 ppb; and xylenes—0.06 ppb) were similar to those from a stationary monitoring site in Deer Park maintained by the TCEQ during 27 April 2018 to 4 May 2018 (benzene—0.19 ppb; ethylbenzene—0.99 ppb; toluene—0.25 ppb; and xylenes—0.05 ppb) [25]. The daily average concentration of PM_2.5_ from eight homes over six weeks in Deer Park was 13 ± 6 µg/m^3^, which is lower than the 2012 National Ambient Air Quality Standard for 24-h PM_2.5_ (35 µg/m^3^) [14]. The results are consistent with PM_2.5_ mass concentrations that we measured between 2013 and 2016 in outdoor air in other locations in Houston that ranged from 11.6 to 13.2 µg/m^3^ [26].

Due to weather-related power outages at several homes in the second week of air monitoring in Deer Park, we obtained full 6-week data of PM_2.5_ and TVOCs from four residential houses out of eight, highlighting the value of battery- or solar-powered sensors for residential monitoring. However, we did not find large differences in the patterns of daily averages of PM_2.5_ and TVOCs when comparing the first two weeks of data to the time series for the entire period. While research-grade instruments accurately measure speciated air pollutants such as BTEX [5,27], they are expensive and require time to analyze the sampled media in a laboratory, which limits their widespread use in disaster response. Direct-reading instruments, including real-time low-cost air sensors, can greatly and rapidly enhance understanding of exposure to VOCs and PM in space and time [26,28,29].

Most participants reported coughing and eye, nose, or throat irritation, which are common health effects associated with short-term air pollution exposure [30,31], particularly PM. During the ITC fire, the mean concentration of PM_2.5_ in Deer Park was 38.5 µg/m^3^ (max = 51.6 µg/m3). Thus, the respiratory symptoms reported in this study may be associated with the short-term peak exposure to PM_2.5_, although PM_2.5_ levels decreased by 63% and reached background levels within a week after the ITC fire.

Only one participant indicated “moderate” depression using the PAQ-8 cut-point score of ≥10 and met the criteria for a provisional diagnosis of depression following major PHQ-8 depression algorithm. While our small sample size precludes drawing conclusions of the health impact of the ITC fire on residents living in surrounding neighborhoods, the relatively low prevalence of PTSD symptoms and depression among participants may suggest a certain “resilience,” which is often found in individuals having prior experience in recovering from a disaster or living in an area frequently affected by disasters [32]. Nonetheless, more than half of participants who reported mild symptoms did indicate they had experienced difficulty at home and work, as well as in relationships with others, during the two weeks after the fire. Given that these are indicators of possible impairment of social and occupational functioning [9,33], future DR2 studies should continue to evaluate mental health as well as physical health impacts, providing findings that can be used to enhance resiliency among residents living in neighborhoods at risk for natural or industrial disasters.

## 5. Conclusions

With operational and knowledge capabilities to carry out disaster assessments, academic institutions must position themselves to lead rapid and localized disaster response activities for ensuring community health and safety [34,35]. However, resource constraints and the reliance on voluntary field staff limit the scope and reach of the response. For example, we limited our pilot study area and promotion activities to a single canvassing event. While this effort produced a modest response, participation among respondents was high (12 out of 13 residents). Given recommendations of ongoing monitoring and reporting of baseline outdoor air quality in neighborhoods near industrial facilities, our efforts and report back represent an important first step in answering residents’ questions about air quality following an industrial disaster, raising environmental health awareness and enhancing community resilience. To our knowledge, the present study is the first published study investigating air quality and the health of affected residents following the ITC fire. Larger and more systematic efforts are needed to address environmental health impacts from future calamitous events, particularly in neighborhoods close to industrial facilities.

## Figures and Tables

**Table 1:** Mean ambient air pollutant levels in Deer Park and Jacinto City on 20 March 2019 (during the ITC Fire) and on 27 March 2019 (5 days after the fire was extinguished).

	Deer Park			Jacinto City		
Pollutant	March 20	March 27	*p*-Value ^a^	March 20	March 27	*p*-Value ^a^
PM_2.5_ (µg/m^3^) ^b^	38.5 ± 4.1	14.4 ± 8.5	<0.001	52.8 ± 6.4	N/A^c^	_
TVOCs (ppb)	79 ± 84	28 ± 40	<0.001	46 ± 69	20 ± 13	0.006
BC (µg/m^3^)	0.90 ± 0.10	0.46 ± 0.15	<0.001	1.30 ± 0.46	0.65 ± 0.33	0.002
UFP (#/cm^3^)	13,575 ± 4408	4895 ± 1566	<0.001	26,888 ± 2275	40,958 ± 4688	<0.001

^a^ Two-tailed *t*-test to evaluate differences in mean levels for each pollutant was conducted between 20 March and 27 March at each location and evaluated at a significance level of 0.05. ^b^ The monitored PM_2.5_ mass from Grimm 11-R was not corrected for a filter-based gravimetric method. ^c^ PM_2.5_ mass data were not recorded due to a battery failure for the Grimm 11-R.

**Table 2:** Summary statistics for residential outdoor levels of fine particulate matter (PM_2.5_) and total volatile organic compounds (TVOCs) measured in Deer Park, TX, between 27 April 2019 and 12 June 2019.

Pollutant	No. of Homes	Mean	Median	Range
PM_2.5_ (µg/m^3^)	8	13 ± 6	12	3–28
TVOCs (ppb)	8	108 ± 98	72	17–536
